# Properties of neurons in the superficial laminae of trigeminal nucleus caudalis

**DOI:** 10.14814/phy2.14112

**Published:** 2019-06-18

**Authors:** Bruno Pradier, Samuel J. McCormick, Ayumi C. Tsuda, Rudy W. Chen, Abigail L. Atkinson, Mollie R. Westrick, Caroline L. Buckholtz, Julie A. Kauer

**Affiliations:** ^1^ Department of Molecular Pharmacology, Physiology & Biotechnology Brown University Carney Institute for Brain Science Providence Rhode Island; ^2^ Present address: Department of Anesthesiology, Intensive Care and Pain Medicine University Hospital Muenster Münster Germany; ^3^ Present address: Department of Psychiatry & Behavioral Sciences Stanford University School of Medicine Stanford California

**Keywords:** Craniofacial, electrophysiological properties, neuronal classification, trigeminal, waveform

## Abstract

The trigeminal nucleus caudalis (TNc) receives extensive afferent innervation from peripheral sensory neurons of the trigeminal ganglion (TG), and is the first central relay in the circuitry underpinning orofacial pain. Despite the initial characterization of the neurons in the superficial laminae, many questions remain. Here we report on electrophysiological properties of 535 superficial lamina I/II TNc neurons. Based on their firing pattern, we assigned these cells to five main groups, including (1) tonic, (2) phasic, (3) delayed, (4) H‐current, and (5) tonic‐phasic neurons, groups that exhibit distinct intrinsic properties and share some similarity with groups identified in the spinal dorsal horn. Driving predominantly nociceptive TG primary afferents using optogenetic stimulation in TRPV1/ChR2 animals, we found that tonic and H‐current cells are most likely to receive pure monosynaptic input, whereas delayed neurons are more likely to exhibit inputs that appear polysynaptic. Finally, for the first time in TNc neurons, we used unsupervised clustering analysis methods and found that the kinetics of the action potentials and other intrinsic properties of these groups differ significantly from one another. Unsupervised spectral clustering based solely on a single voltage response to rheobase current was sufficient to group cells with shared properties independent of action potential discharge pattern, indicating that this approach can be effectively applied to identify functional neuronal subclasses. Together, our data illustrate that cells in the TNc with distinct patterns of TRPV1/ChR2 afferent innervation are physiologically diverse, but can be understood as a few major groups of cells having shared functional properties.

## Introduction

The trigeminal pathway is critically involved in mediating craniofacial pain, and consists of peripheral sensory neurons with cell bodies in the trigeminal ganglion (TG) and projections to both peripheral tissues and central brain regions. Despite the prevalence of orofacial pain conditions, including primary headache, trigeminal neuralgia, and trigeminal inflammatory or neuropathic pain, little is known about the cell type distribution or circuitry of central neurons in the trigeminal nucleus (Gobel 1978; Sedlacek et al. 2007; Davies and North 2009). This brainstem nucleus is subdivided along its rostro‐caudal axis into the trigeminal nucleus oralis, interpolaris, and caudalis, with the caudal part (TNc, SP5C, or medullary dorsal horn) receiving predominantly nociceptive afferents. The TNc receives synaptic inputs mostly from small diameter fibers of the TG that innervate various tissues including skin, meningeal dura, tooth pulp, or cornea (Hargreaves 2011). It is the first central relay in the orofacial pain pathway and, similar to the spinal dorsal horn, is made up of a heterogeneous neuronal population that comprises primarily excitatory and inhibitory interneurons, with a smaller population of projection neurons found in lamina I and V (Gobel 1978; Villanueva and Noseda 2013). TNc neurons receive peripheral nociceptive information through C‐ and A*δ*‐fibers that terminate extensively in laminae I and II (Hamba and Onimaru 1998; Pradier et al. 2018). They also receive synaptic innervation from local interneurons (Pradier et al. 2018) and from long distance projections of top‐down pain‐regulating afferents (e.g., from the RVM, cortex, hypothalamus) (Noseda et al. 2010; Abdallah et al. 2013; Robert et al. 2013; Brennan and Pietrobon 2018; Wang et al. 2018).

Recent studies characterizing the electrophysiological properties of TNc laminae I/II neurons defined four cell types exhibiting a tonic, phasic, or delayed action potential (AP) firing pattern, but also single‐spiking neurons (Sedlacek et al. 2007; Davies and North 2009; Alba‐Delgado et al. 2015). While studies in the dorsal horn of the spinal cord found that firing pattern does not always correlate well with inhibitory or excitatory cell identity (Yasaka et al. 2010), the use of cre‐dependent fluorescent reporter mouse lines has been shown to be especially useful (Heinke et al. 2004; Punnakkal et al. 2014; Alba‐Delgado et al. 2015; Peirs et al. 2015; Cheng et al. 2017; Moehring et al. 2018) to correlate neurophysiological with neurochemical properties. Here we report on the basic electrophysiological properties of 535 superficial lamina I/II TNc neurons acquired through whole‐cell patch‐clamp recordings. Based on their firing pattern, we assigned these cells to five main groups, including (1) tonic, (2) phasic, (3) delayed, (4) H‐current, and (5) tonic‐phasic neurons. We have also recorded from genetically identified and fluorescently labeled neurons to test for correlations with basic electrophysiological properties. Finally, we used an unsupervised clustering approach to test whether this method might produce similar or distinct cell groups.

## Methods

### Animals

All animal procedures were approved by the Institutional Animal Care and Use Committee of Brown University, Providence. Transient receptor potential vanilloid receptor 1 (Trpv1)‐Cre, vesicular GABA transporter (VGAT)‐Cre, somatostatin (SOM)‐cre, cyclin E2 (CCNE2)‐GFP, lox‐STOP‐lox‐ChR2‐EYFP, and lox‐STOP‐lox‐TdTomato mice were purchased from The Jackson Laboratory. Trpv1‐Cre^+/+^ mice were mated with ChR2‐EYFP^+/+^ mice to generate Trpv1^+/−^/ChR2‐EYFP^+/−^ offspring (referred to as TRPV1/ChR2). TRPV1/ChR2 mice used in this study were first‐generation progeny of homozygous parents. Vgat‐cre^+/+^ and Som‐cre^+/+^ were mated with TdTomato^+/+^ mice to generate Vgat‐cre^+/+^/TdTomato^+/+^ and Som‐cre^+/+^/TdTomato^+/+^ (referred to as VGAT‐ and SOM/TdTom, respectively). CCNE2‐GFP^+/+^ mice were kept on a homozygous breeding schedule to generate CCNE2‐GFP^+/+^ offspring. Both male and female mice were used for this study.

### Preparation of brainstem slices

Electrophysiological recordings were performed as recently described (Chirila et al. 2014; Pradier et al. 2018). In brief, under deep anesthesia brainstems of p14 – p35 mice were removed after cardiac perfusion with 34°C pre‐warmed and oxygenated ACSF (in mM: NaCl 125; NaHCO_3_ 26; glucose 25; MgCl_2_ 6; KCl 2.5; CaCl_2_ 1.5; NaH_2_PO_4_ 1.25; kynurenic acid 1). Then, two to three 250‐*μ*m thick coronal brainstem slices were cut on a vibratome (Leica 1200) starting approximately 0.5 – 0.6 mm caudal to the obex to yield the trigeminal nucleus caudalis (TNc). Slices were allowed to rest at 34°C in ACSF for 1 h before they were kept at RT until recording.

### Whole‐cell recording

TNc slices were transferred into a recording chamber, in which slices were continuously perfused with 28–30°C warm ACSF (in mM: NaCl 119; NaHCO_3_ 26; glucose 25; CaCl_2_ 4; MgCl_2_ 4; KCl 2.5; Na‐ascorbate 1.3; NaH_2_PO_4_ 1) bubbled with 95%O_2_/5%CO_2_; 100 *μ*mol/L picrotoxin was used to block glycine and GABA_A_ receptors. Patch pipettes (5–7MΩ) were pulled from borosilicate glass (Sutter Instruments) and filled with a K‐gluconate‐based internal recording solution (in mM: K‐gluconate 117, NaCl 2.8, MgCl_2_ 5, CaCl_2_ 0.2, HEPES 20, Na‐ATP 2, Na‐GTP 0.3, EGTA 0.6 and 0.2% biocytin). Reported membrane potential values were not corrected for the liquid junction potential, which was experimentally determined to be 8 mV under our recording conditions. To record synaptic inputs from TRPV1/ChR2 animals, an optic fiber (230 *μ*m diameter, Plexon) was placed at the dorsolateral slice edge to stimulate TRPV1/ChR2 primary afferents with light (Plexon LED, 465 nmol/L, 0.5–1 msec, 0.1–10mW). Cells from lamina I/II were identified visually and recorded using whole‐cell patch‐clamp. For synaptic recordings, neurons were voltage‐clamped at −70 mV. Excitatory postsynaptic currents (EPSCs) were optically evoked with a LED (Plexon, 465 nm, 1–10mW) and stimulated every 30 sec.

### Intrinsic properties

Resting membrane potentials (RMP, in mV) were measured within 1‐2 min of break‐in to the whole‐cell configuration, and all intrinsic properties were measured within 5 minutes of break‐in as well. Input resistances (R_m_, in MΩ) were calculated by measuring the voltage response, from rest, following a negative current injection (25 pA; 300 msec duration). Membrane time constants (*τ*_m_, in ms) were determined by fitting a single exponential curve to responses following 25pA current steps. Membrane capacitance (C_m_, in pF) was read from the membrane test in pClamp's Clampex program. Rheobase (in pA) was defined as the minimal positive current injection needed to evoke an action potential (AP) from a holding potential of −70 mV (1000 msec; 0–1000 pA current ramp injection). Following current injections of 300 msec in 25 pA increments, AP amplitudes (in mV) were determined for the first AP evoked by rheobase current and measured as the difference between the threshold and the peak of the AP. For inclusion in the data set, neurons had to have stabile resting membrane potential of at least ‐40 mV (−48 mV accounting for the liquid junction potential) and an action potential that overshot 0 mV. AP half‐widths (in msec) were determined for the first AP evoked by rheobase current and measured at the half height between threshold and the peak of the AP. AP latencies (in ms) were determined by measuring the time from the start of the rheobase current pulse to the peak of the first AP. Spike frequency adaptation was determined by calculating the adaptation ratio, defined as the firing frequency at the last interspike interval divided by the firing frequency at the first interspike interval during a 200 pA (300 msec) current step. Fast after‐hyperpolarization potentials following the first AP evoked by rheobase current (fAHP, in mV) were measured as the difference between the AP threshold and the minimum membrane potential attained during the fAHP. The AP rise time and decay time (in msec) were measured as the time needed to reach 10–90% (rise) or 90–10% (decay) of the AP amplitude. Amplitude (in mV) of AHPs occurring within 500 msec following the end of a depolarizing 200 pA current injection was measured as the difference between resting membrane potential and the peak of the AHP (AHP, in mV). Peak sag amplitudes (in mV) were measured in response to a 300 msec negative current step (−25 pA) and calculated relative to the steady‐state voltage at the end of the step (average of last 100 msec). Additionally, in some recordings, peak sag‐currents were measured in voltage clamp evoked by a voltage step from −50 mV to −100 mV. Analysis of electrophysiological data was carried out using Molecular Devices Clampfit 10 and Clampex 10, MatLab, Prism, and Microsoft Excel.

### Classification of neuronal cell types

We classified 535 neurons according to their discharge pattern induced by depolarizing current injections as reported recently (Sedlacek et al. 2007; Davies and North 2009), and identified five different groups. (1) Tonic firing cells showed AP discharge throughout the 300 ms current injections. (2) Delayed firing neurons had a delayed onset to spike and a hyperpolarizing notch during the depolarization phase after the start of a depolarizing current injection. To be classified as a delayed cell, we required the AP latency to be at least 30 ms upon an injection of 200 pA. (3) Phasic neurons ceased spiking in the second half of the current injection or showed only an initial burst of action potentials. (4) Characteristic of H‐current cells was the coincidence of a sag‐potential or current (>25pA) and an AHP (2.5–19 mV) that peaked ~55 msec following termination of a 200pA depolarizing current; however, neurons were assigned to the H‐current group based on these properties regardless of their firing pattern. (5) Tonic‐phasic cells showed tonic discharge pattern with low depolarizing currents (100pA) and an accommodating phasic discharge pattern following high (200 pA) depolarizing currents.

### Synaptic properties

Optically evoked excitatory postsynaptic currents (EPSCs) were recorded in the presence of picrotoxin to block GABA_A_ and glycine receptor‐mediated conductances. In some cells, optical activation could evoke EPSCs in cells of lamina I/II that appeared monosynaptic (short latency, low jitter), but in other cells often evoked apparently polysynaptic EPSCs as well. Measurement of the kinetics of monosynaptic EPSCs included: latency (in ms, measured as the time from light pulse to EPSCs onset), rise time (in ms, representing the time from EPSC onset to peak for 10–90% of the event amplitude), and decay time constant (*τ*−decay, in msec, were taken from a single exponential curve fitted from peak to baseline). For minimal synaptic stimulation, the LED‐driver was triggered with 1 msec pulses of increasing voltages (0–5V; 0.1–10mW) in 0.1V increments. Amplitudes of EPSCs elicited at the lowest light intensity were referred to as minimal EPSCs (in pA).

### Immunohistochemistry

Following whole‐cell patch‐clamp recording, some of the slices were fixed in 4% paraformaldehyde at 4°C for up to 4 days. Slices were then washed in PBS in 0.2% PBS‐T for 30 min, followed by a permeabilization in pre‐warmed (60–80°C) 1% PBS‐T. After a 10 min wash in 0.2% PBS‐T, slices were incubated for 48–72 h in 1:500 Alexa Fluor 488‐conjugated streptavidin in a moist compartment at 4°C to reveal biocytin‐labeled neurons. The next day, slices were washed three times before mounting in Fluoromount‐G^™^. Sealing with water varnish prevented sections from drying out. Occasionally, Alexa647‐conjugated isolectin B4 (1:200, IB4, Molecular Probes Cat# I21411, RRID:AB_2314662) was used to label terminals of non‐peptidergic neurons in TNc slices.

### Morphological analysis

Images were acquired on a Zeiss LSM 800 confocal microscope using a 40× water immersion objective or a 20× objective at a 1024 × 1024 resolution. For the morphological analysis, individual optical sections of confocal images that included cell body, axon, and dendrites were superimposed in Adobe Photoshop and all profiles were labeled, selected, and pasted onto a black background as previously described (Yasaka et al. 2010).

### Clustering analyses

An unbiased classification of superficial TNc neurons was based on an unsupervised cluster analysis using eight electrophysiological parameters (including AP amplitude, AP half‐width, AP rise and decay time, rheobase, latency to first AP, fAHP, and AHP) from 491 neurons recorded. Clustering was implemented with a hierarchical cluster analysis using Ward's method (Ward 1963) in SPSS (IBM) software package. We then plotted the squared Euclidian distance as a function of cluster stage (inset Fig. 6). Using this agglomeration schedule, we determined the best number of clusters to be 5 since the final large decrease in linkage distance occurred between stages 4 and 5. The dashed line indicates the cutoff in the dendrogram (Fig. 6).

For the spectral clustering of spike wave forms (Luxburg 2007), we first isolated traces of rheobase APs, using depolarizing current steps from 25 to 200 pA. The dV/dt of the membrane potential prior to the AP threshold and following the fAHP appeared to differ on a cell by cell basis, and we therefore created a 90‐msec spike sample by including 40 msec before and 50 msec after the AP peak along with the AP (see Fig. 7A). Next, rheobase spikes were isolated from each trace and 50 ms of the AHP was concatenated to this spike form. Using this approach required us to remove some cells from the analysis: cells were excluded if the rheobase AP appeared within 40 msec of onset or 50 msec before the end of the depolarizing current, or if multiple APs occurred within 50 msec of the rheobase spike.

We adapted the method of spectral clustering (Luxburg 2007) to find inherent structures in our samples of rheobase spike traces. Spectral clustering involves computing a similarity matrix A and using the spectrum (eigenvalues) of that matrix in order to reduce the dimensionality of the associated clustering problem. Specifically, the eigenvectors associated with the smallest eigenvalues of the graph Laplacian matrix of A are computed, and the standard k‐means algorithm is applied to this lower‐dimensional embedding in order to separate the data points into clusters. To implement spectral clustering, we had to choose an appropriate similarity metric that would capture the structural patterns in the data that we believed to be significant. Preprocessing the data according to the concatenation methodology described above allowed us to extract what we believed were the most significant portions of the waveform. After aligning the resulting samples, we calculated the Euclidean distance between all pairs of samples in the data set. That is, the i, jth entry of A (A_ij_) was computed as the Euclidean distance between preprocessed data samples i and j (Nguyen et al. 2014). We then used the spectral clustering implementation in the Python package “scikit‐learn” to determine the cluster assignments.

### Statistical analysis

All results are expressed as the mean ± S.E.M. All statistical analyses were carried out using GraphPad Prism^®^ software. One‐way ANOVA followed by Tukey's post hoc test was used to analyze data sets unless otherwise noted. Using large data sets enables detection of even small differences, which are statistically significant but are unlikely to have physiological meaning. In Table 1, we therefore report the effect sizes in addition to p‐values alone. These were calculated using the following formula: (Mean (group 1) – Mean (group 2))/standard deviation (group 1 + 2) (Sullivan and Feinn 2012). Classically, the relative effect size is considered medium with values between 0.5 and 0.7 and large for values > 0.8. Statistical tests were considered to be significant at a 95% confidence interval, with *P*‐values reported in the results section.

**Table 1:** Results of one‐way ANOVA followed by Tukey multiple comparisons for neuronal properties

	Property	ANOVA		Multiple comparisons (*P*‐values; effect size)																			
		*P*‐value	*f*‐values	D‐T		D‐P		D‐H		D‐TP		T‐P		T‐H		T‐TP		P‐H		P‐TP		H‐TP	
Active properities	Amplitude	< 0.0001	*F* (4, 480) = 21.5	****	0.90	****	0.63	ns	0.28	****	0.89	*	0.40	****	0.69	ns	0.01	ns	0.37	ns	0.38	**	0.64
	Half‐width	< 0.0001	*F* (4, 480) = 55.2	**	0.45	****	0.83	****	1.09	ns	0.26	****	1.17	****	0.93	***	0.76	****	1.45	***	0.61	****	1.21
	Rise time	< 0.0001	*F* (4, 458) = 50.9	****	1.08	ns	0.17	****	1.18	ns	0.26	****	1.24	ns	0.56	****	1.04	****	1.29	*	0.43	****	1.07
	Dec. time	< 0.0001	*F* (4, 464) = 58.8	ns	0.06	****	1.18	**	0.69	****	0.87	****	1.18	***	0.75	****	0.84	****	1.36	***	0.53	****	1.14
	Lat.to first	< 0.0001	*F* (4, 480) = 112.5	****	1.33	****	1.42	****	1.62	****	1.34	ns	0.37	****	0.94	ns	0.07	**	0.62	ns	0.28	***	0.80
	fAHP	< 0.0001	*F* (4, 480) = 39.9	ns	0.23	****	1.24	****	0.99	****	1.05	****	1.06	****	0.80	****	0.81	ns	0.22	ns	0.33	ns	0.08
	Adap. Index	< 0.0001	*F* (4, 385) = 62.9	****	1.37	****	1.00	****	1.44	**	0.62	*	0.60	*	0.49	****	1.14	****	0.76	ns	0.53	****	1.11
	Rheobase	< 0.0001	*F* (4, 478) = 87.2	****	1.20	****	1.48	ns	0.03	****	1.47	****	0.84	****	1.03	**	0.80	****	1.30	ns	0.01	****	1.11
Passive properties	sag	< 0.0001	*F* (4, 477) = 30.1	ns	0.06	ns	0.15	****	1.00	ns	0.06	ns	0.14	****	1.21	ns	0.01	****	1.13	ns	0.11	****	0.93
	AHP	< 0.0001	*F* (4, 480) = 121.5	ns	0.08	ns	0.17	****	1.84	*	0.56	ns	0.21	****	1.77	ns	0.40	****	1.72	**	0.64	****	1.67
	t	< 0.0001	*F* (4, 461) = 29.3	ns	0.16	ns	0.10	****	1.31	ns	0.43	ns	0.26	****	1.30	ns	0.24	****	1.38	*	0.57	****	1.52
	R_m_	< 0.0001	*F* (4, 480) = 36.2	ns	0.16	****	0.57	****	1.25	****	0.79	****	0.67	****	1.05	****	0.87	****	1.35	ns	0.25	****	1.33
	Cap	< 0.0001	*F* (4, 479) = 17.2	**	0.40	***	0.65	ns	0.02	*	0.63	****	0.86	*	0.35	****	0.81	*	0.49	ns	0.03	ns	0.42
	RMP	< 0.0001	*F* (4, 456) = 7.2	ns	0.09	***	0.52	**	0.68	ns	0.23	**	0.45	**	0.61	ns	0.15	ns	0.08	ns	0.28	ns	0.42
								*P < 0.05				**P < 0.01				***P < 0.001				****P < 0.0001			

*P*‐ and *F*‐values of ANOVA (left); *P*‐values (in asterisks) and effect sizes for each comparison (right). Only large effect sizes (>0.8) are highlighted in green. (f)AHP, (fast) after‐hyperpolarizing potential; RMP, resting membrane potential; dec time, decay time; *τ*_m_, membrane time constant; R_m_, input resistance; C_m_, capacitance; T, tonic; D, delayed; P, phasic; H, H‐current; TP, tonic‐phasic. *P*‐values are given in asterisks; *P < 0.05; **P > 0.01; ***P > 0.001; ****P > 0.0001. Effect sizes are considered small (0.2–0.4), medium (0.5–0.7), or large (>0.8).

## Results

### Five prominent cell types in superficial laminae of the TNc

**Figure 1:**
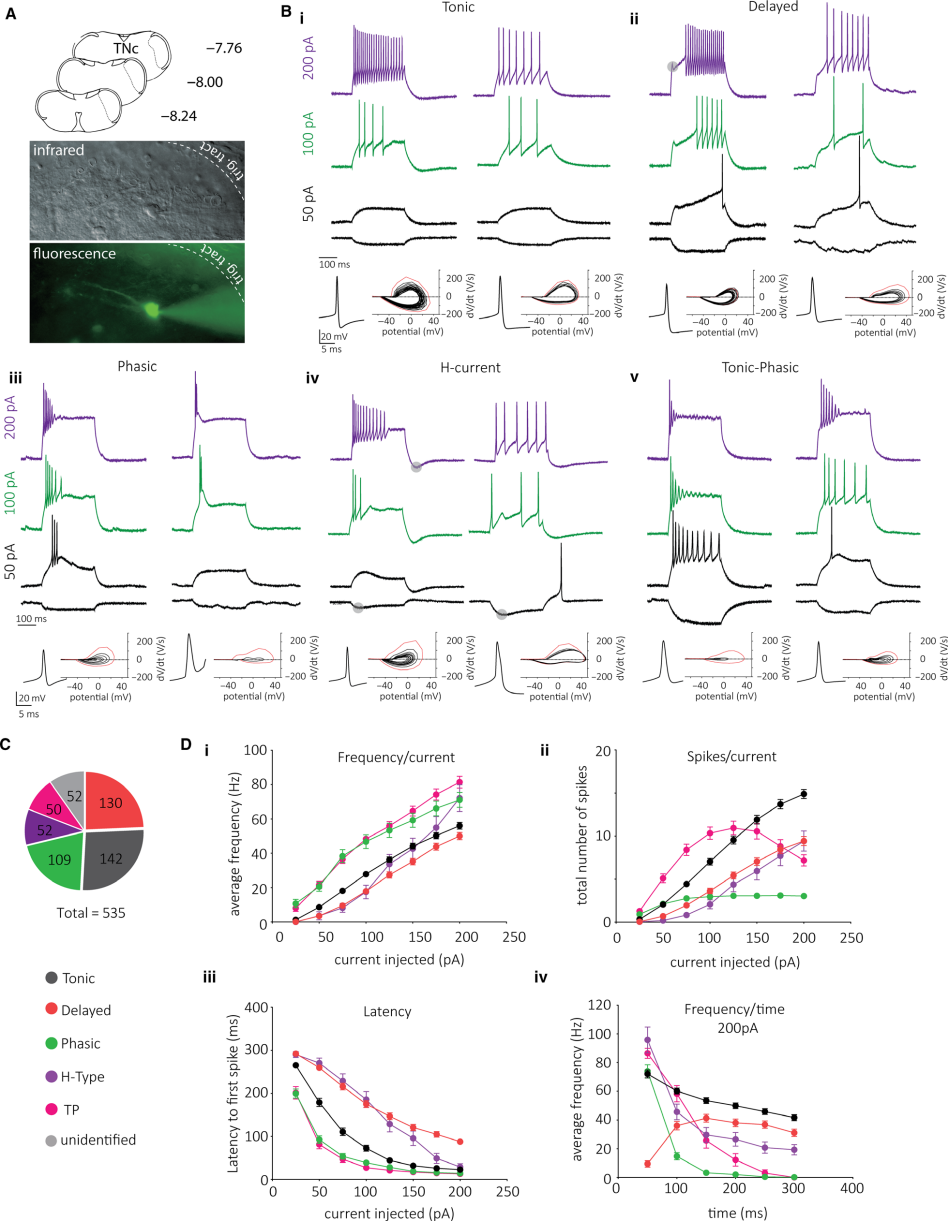
AP discharge patterns of lamina I/II neurons in the TNc. (A) Schematic coronal (transverse) slices of the TNc with stereotaxic coordinates on the left illustrating the recording sampling site (dotted lines, top). Live infrared and fluorescent images of a lucifer‐yellow filled cell (A, bottom). (B) Firing properties of five classes of TNc neurons with two examples per group. Example voltage responses are shown for ‐25 (black), +50 (black), +100 (green), and + 200 (purple) pA current injections for 300 msec (top); (i) tonic, (ii) delayed; (iii) phasic, (iv) H‐current, and (v) tonic‐phasic neurons. Rheobase APs and phase plot of APs elicited following a 200pA depolarizing current (bottom). (C) Pie chart of frequency of each cell type (including unidentified cell types) showing absolute numbers. (D) Plots of spike frequency (i), spike number (ii), and latency (iii) as a function of current injected and frequency as a function of time for a 200pA depolarizing current injection (iv) for each of the five groups. Color code for (D) is same as in (C).

We recorded from neurons located in superficial laminae I and II in the TNc (Fig. 1A) and manually assigned each neuron to groups depending on their spiking pattern (Fig. 1B), similarly to previous reports (Sedlacek et al. 2007; Davies and North 2009). We found five prominent groups, with tonic (1), delayed (2), and phasic (3) firing neurons being the most prevalent. We also grouped neurons we termed H‐current cells (4) that showed a prominent sag‐potential (or sag‐current) and a fast peaking AHP regardless of the firing pattern. We identified a further subgroup that would considerably change its spiking pattern the more we depolarized it. Depending on the amount of current injected, these neurons switch from a tonic (100pA) to a phasic (200pA) firing pattern and we thus named these tonic‐phasic (TP) cells (5). From 535 recorded neurons, we found 26% tonic, 24% delayed, 20% phasic, and 9% each of H‐current and TP. The remaining 12% contained less frequently encountered cell types that include single‐ (1%) or gap‐ (4%) firing neurons, but also rare and unidentified cell types (Fig. 1C).

### Electrophysiological properties of different cell types

We then plotted the averaged spike frequency, number of spikes, and latency to first spike as a function of levels of injected current (Fig 1Di–iii). Tonic firing cells have a low‐to‐intermediate spiking frequency range compared to other cell types while at the same time the highest total spike count over a 300 ms depolarization. Delayed cells show the lowest average frequency, intermediate number of spikes, and (due to the delayed onset of AP firing) the longest latency to the first spike. We next probed the voltage‐dependence of presumed A‐type K^+^ currents in delayed cells. Consistent with the presence of A‐type currents, holding cells at depolarized membrane potentials significantly decreased the latency to first spike and application of 4‐AP (1.5 mmol/L) led to a change from delayed onset to fast‐onset spiking pattern (data not shown) similar to what has been described for delayed cells in the spinal cord (Yoshimura and Jessell 1989).

Phasic neurons tend to show burst firing and thus have the highest average frequency, but at the same time the lowest number of spikes over a 300 ms depolarization. Together with TP cells, phasic neurons have the shortest latency to spike. TP cells were found to have the highest average frequency and greatest number of spikes at low injected current levels. Because of their phenotypic switch (tonic‐>phasic), however, the number of spikes is strongly reduced at higher levels of injected current.

H‐current cells exhibit low spike frequency during small current injections (probably due to their high rheobase), which sharply increases with higher levels of injected current. The spike count of H‐current cells lies at an intermediate level, perhaps because different firing patterns can be observed in neurons with H‐current. As the coincidence of sag‐potential and AHP was prominent in H‐current cells, we ran a correlation analysis and found that the higher the I_h_ current the larger the AHP amplitude (*R* ^2^ = 0.22, *P* = 0.012). We then tested in two neurons whether the sag‐current and the AHP were modulated by blocking hyperpolarization‐activated channels (HCN) with ZD 7288 (10 *μ*mol/L). Indeed, we found in two cells that 10 min application of ZD 7288 blocked I_h_ currents, but surprisingly also the AHP (data not shown).

Plotting the spiking frequency of these groups over time bins of 50 ms shows that phasic and TP cells quickly run toward zero, while delayed cells have a late onset of firing activity, and together with tonic cells show the lowest level of frequency adaptation (Fig 1Div).

**Figure 2:**
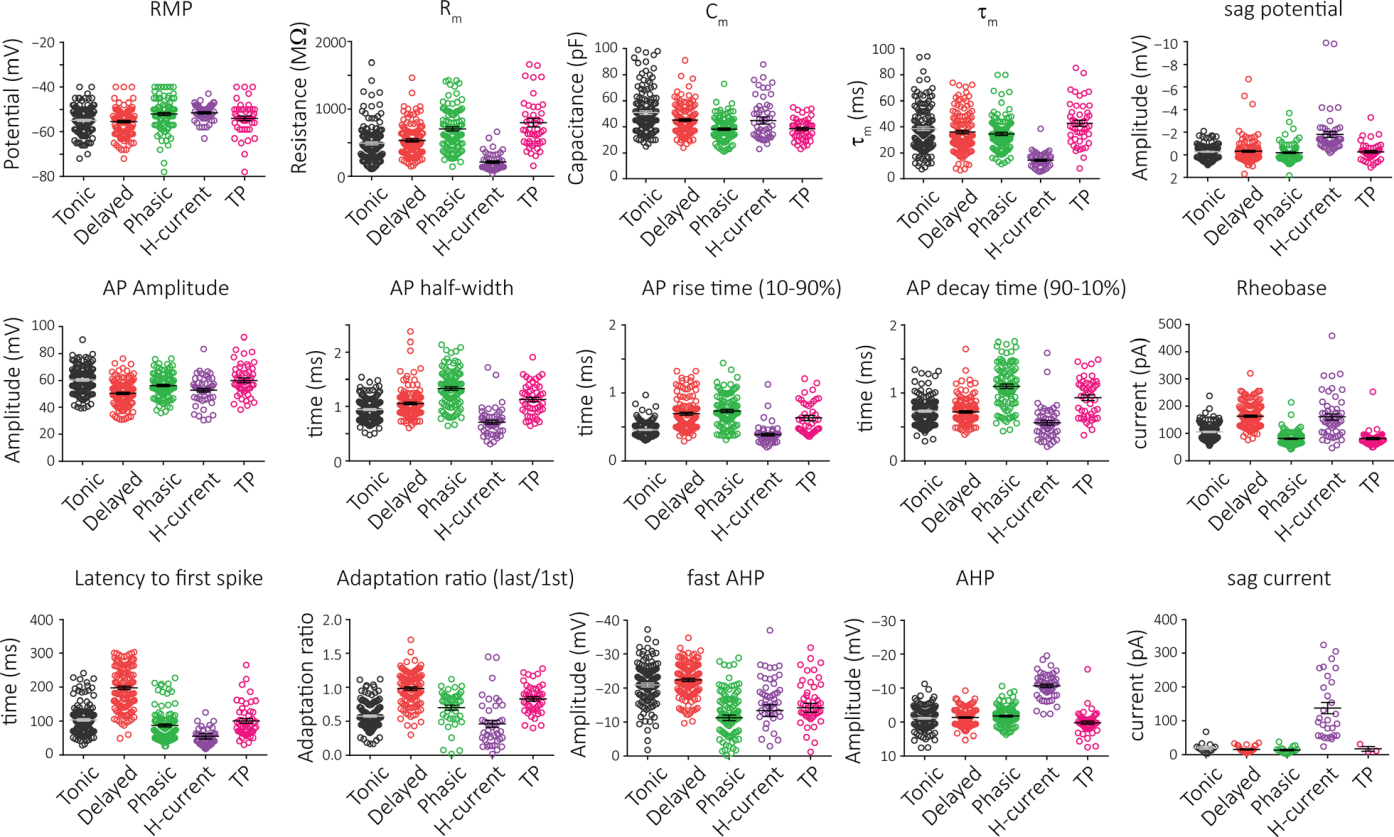
Electrophysiological properties of lamina I/II neurons in the TNc. Scatter dot plots of the recorded neurons for each property, further showing mean ± S.E.M. RMP: resting membrane potential, R_m_: input resistance, C_m_: input capacitance, *τ*_m_: membrane time constant, AP: action potential, AHP: after‐hyperpolarizing potential.

An overview of the electrophysiological properties of these neuronal subgroups is shown in Figure 2. Of note are H‐current neurons that stand out for their group‐defining properties (large sag and AHP), but also for their fast time constant, fast spike kinetics (i.e., narrow half‐width, fast AP rise, and decay time), and high rheobase. In contrast, phasic neurons tend to have broad spikes with a particularly long decay time. Tonic neurons have the greatest spike amplitude and considerably fast spike kinetics. Similarly, TP cells also have very fast spike kinetics. On the other hand, these neurons also share properties with phasic neurons; having a low rheobase and a small fAHP makes them easily excitable and allows for high‐frequency firing. Characterized by their long latency to spike, delayed neurons have the highest rheobase and the smallest spike amplitude with a rather slow AP rise time. Table 1 presents a detailed summary of these electrophysiological parameters and pairwise statistical comparisons between cell types along with the effect size. With this comparison, we found the largest effect sizes with active properties. Interestingly, phasic and TP cells appeared to show the least differences. It should be noted that our cells and their classification were made under one set of circumstances, and as the properties of cells in the trigeminal nucleus as in the dorsal horn can change in response to synaptic drive, injury, and inflammation. It will be of interest to see how these groups change in different conditions.

### Kinetics of synaptic currents evoked by optical TRPV1/ChR2 afferent stimulation

**Figure 3:**
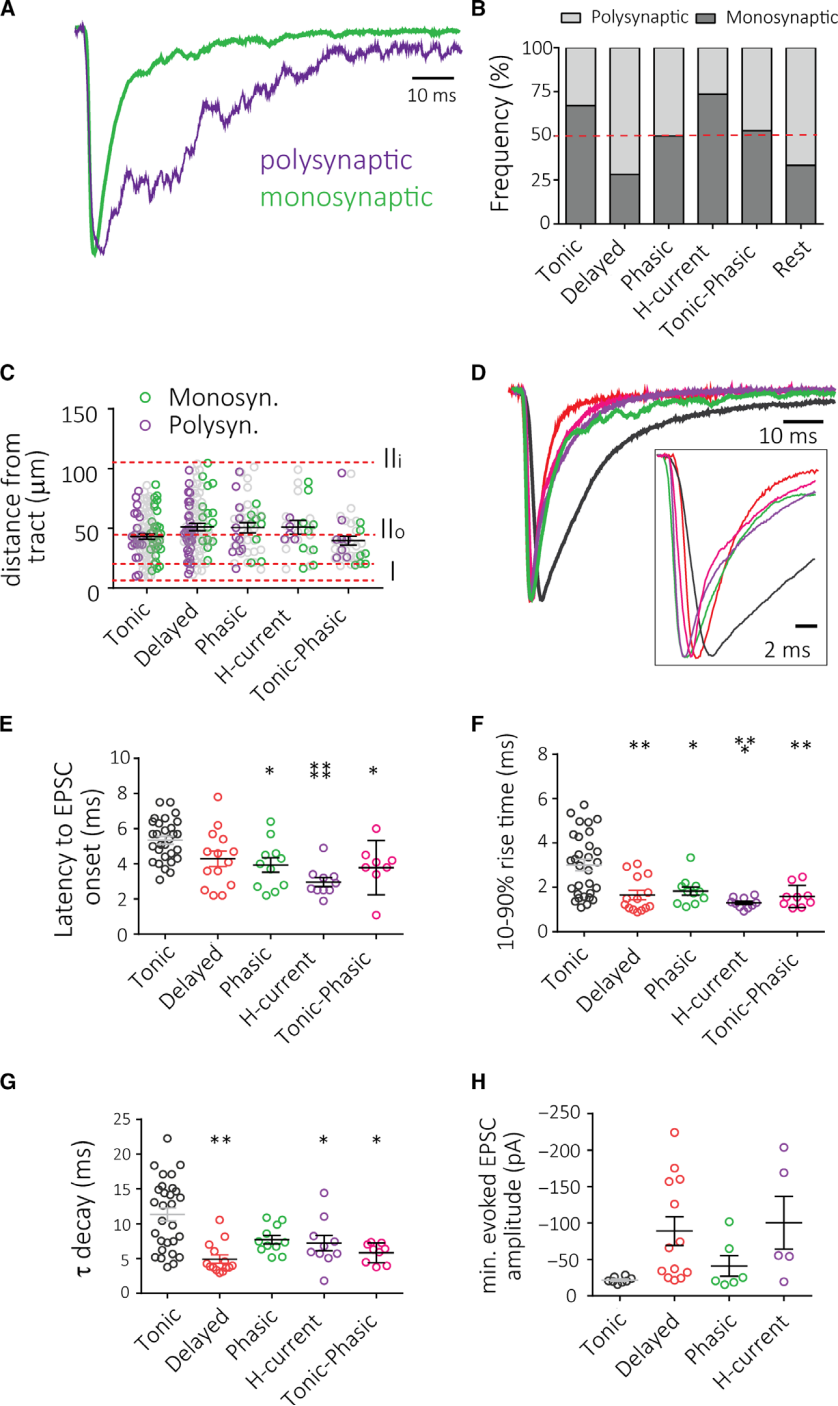
Synaptic properties of TRPV1/ChR2 inputs onto superficial laminae TNc neurons. (A) Example traces for apparently monosynaptic (green) or polysynaptic (purple) EPSCs from different cells. (B) Observed frequency of mono‐ and polysynaptic inputs for each cell type in percent. (C) Distance of recorded cells form trigeminal tract separated by cell type (gray) showing mean ± S.E.M. Cells receiving monosynaptic (green) or polysynaptic (purple) input are highlighted separately. (D) Representative example traces for monosynaptic EPSCs for tonic (black), delayed (red), phasic (green), H‐current (purple), and TP (pink) cells. (E‐GF) Kinetics of monosynaptic EPSCs including latency to onset of EPSCs (E), as well as rise time (F) and decay time constant (G) for each cell type. (H) EPSCs amplitudes induced by the minimal amount of light necessary to evoke any synaptic response (0.1–10mW, 1 msec). Asterisks indicate statistically significant post hoc comparison with tonic cells. *P < 0.05, **P < 0.01, ***P > 0.001, ****P < 0.0001.

To test the differences among cell classes with respect to afferent input, we optogenetically drove primary trigeminal afferents that expressed TRPV1/ChR2 to characterize evoked EPSCs in each cell type (Pradier et al. 2018). In some of the neurons, even large light‐evoked currents appeared monosynaptic (short latency, little jitter, simple kinetics), but in many neurons light‐evoked multiple overlapping EPSCs, most likely induced by feed‐forward excitation producing polysynaptic EPSCs (Fig. 3A), referred here as “polysynaptic”. We next investigated how frequently poly‐ versus monosynaptic TRPV1/ChR2 inputs would occur in each of the cell groups defined in Figures 1 and 2 (Fig. 3 and Table 2). Interestingly, we found that tonic and H‐current cells are far more likely to receive monosynaptic rather than polysynaptic TRPV1/ChR2 input, and in contrast, delayed neurons are more likely to receive polysynaptic TRPV1/ChR2 inputs (Fig. 3B). While the ratio of mono‐ versus polysynaptic input was not changed in phasic neurons, we noted significantly reduced AP firing in those receiving polysynaptic innervation and confirmed that nine of 15 phasic with polysynaptic neurons showed burst firing compared to three of 15 receiving monosynaptic input. We next investigated the laminar localization of cell types in the TNc (Fig. 3C). Generally, tonic and delayed cells were present in lamina I and II, whereas phasic, H‐current, and TP cells were predominantly found in lamina II (gray circles). Separating cells by input type, it appears that mono‐ and polysynaptic‐EPSC shapes show a similar laminar distribution, with the exception of delayed cells that showed more cells receiving polysynaptic input in lamina I and IIo (Fig. 3C).

**Table 2:** Statistical comparison of cell types receiving either mono‐ or polysynaptic input. Values on the top of each row represent average, followed by ±S.E.M. (middle) and the p‐value of a t‐test (bottom)

Celltype	D		T		P		H		TP	
Input	Mono	Poly	Mono	Poly	Mono	Poly	Mono	Poly	Mono	Poly
n	16	41	49	23	15	15	14	5	9	8
AP Amplitude (pA)	48.3	51.4	60.5	58.3	55.3	56.5	57.0	51.5	57.5	64.8
	2.2	2.8	1.4	1.3	2.7	2.2	1.4	1.3	2.9	4.3
	0.335		0.357		0.745		0.055		0.188	
AP Half‐width (ms)	1.0	1.1	1.0	1.0	1.2	1.3	0.8	0.6	1.1	1.2
	0.0	0.1	0.0	0.0	0.1	0.1	0.0	0.1	0.1	0.1
	0.509		0.089		0.580		0.204		0.292	
AP Rise time (ms)	0.7	0.7	0.5	0.5	0.7	0.7	0.4	0.4	0.6	0.7
	0.1	0.1	0.0	0.0	0.1	0.1	0.0	0.0	0.1	0.1
	0.852		0.168		0.962		0.804		0.435	
AP Decay time (ms)	0.7	0.7	0.7	0.8	0.9	1.0	0.6	0.4	0.8	1.0
	0.0	0.0	0.0	0.0	0.1	0.1	0.0	0.0	0.1	0.1
	0.838		0.291		0.509		0.048		0.113	
Rheobase (pA)	168.6	157.3	115.2	97.4	90.0	77.8	151.5	139.4	81.2	75.8
	11.6	10.7	4.1	3.7	5.3	3.2	14.5	13.9	2.6	7.7
	0.385		0.010		0.059		0.671		0.513	
Latency (200pA, ms)	89.5	89.2	22.8	16.8	17.3	11.4	21.8	13.6	13.1	16.0
	13.8	14.0	1.3	1.0	2.3	0.7	2.9	0.9	1.2	3.4
	0.987		0.005		0.022		0.119		0.446	
No. of spikes (200pA)	9.4	8.1	13.0	14.3	4.9	2.1	8.6	14.6	7.1	4.3
	1.3	1.5	0.6	1.0	0.8	0.2	1.3	2.0	1.8	0.5
	0.446		0.319		0.002		0.061		0.164	
fAHP (mV)	23.9	21.4	22.2	23.2	13.7	12.8	16.7	22.1	16.3	16.7
	2.4	1.1	0.7	0.7	2.6	1.6	1.4	0.8	5.5	1.7
	0.174		0.338		0.781		0.038		0.949	
Adaptation index	1.1	1.0	0.6	0.6	0.7	0.8	0.3	0.6	0.8	0.9
	0.1	0.1	0.0	0.0	0.0	0.0	0.0	0.1	0.0	0.1
	0.409		0.584		0.289		0.006		0.389	
AHP (mV)	‐1.1	‐1.3	‐1.2	‐0.1	‐1.9	‐2.6	‐10.2	‐10.0	2.3	0.6
	0.6	0.5	0.3	0.5	0.8	0.7	0.8	0.4	1.0	0.6
	0.804		0.084		0.527		0.866		0.202	
C_m_ (pF)	43.2	48.1	59.0	45.0	43.2	37.5	53.4	45.0	37.0	35.1
	1.7	3.3	2.8	1.5	2.8	2.5	4.6	3.5	2.0	2.0
	0.164		0.002		0.135		0.343		0.528	
RMP (mV)	‐57.1	‐55.7	‐56.9	‐55.9	‐50.6	‐54.4	‐45.4	‐51.4	‐54.4	‐50.9
	1.3	3.6	0.8	0.9	1.9	1.9	7.8	0.8	1.8	1.3
	0.487		0.603		0.169		0.656		0.141	
R_m_	472.4	582.9	407.7	633.2	546.4	673.2	195.2	272.5	889.3	663.4
	55.4	63.4	27.7	51.2	64.5	77.2	27.5	20.2	83.7	118.0
	0.132		0.001		0.218		0.145		0.719	
*τ*_m_ (ms)	32.2	41.9	35.8	44.0	32.0	36.3	15.5	17.2	50.2	45.0
	3.2	4.3	1.7	3.1	3.2	4.5	1.0	0.8	5.3	5.4
	0.047		0.060		0.444		0.426		0.538	
sag‐potential (mV)	‐0.2	‐0.2	‐0.2	‐0.1	‐0.3	‐0.3	‐1.2	‐1.5	0.3	‐0.5
	0.1	0.3	0.1	0.1	0.1	0.1	0.1	0.2	0.1	0.1
	0.806		0.751		0.643		0.248		0.001	
Dist. to tract (*μ*m)	65.5	52.3	50.6	49.4	54.1	54.1	52.6	52.6	36.5	50.0
	7.1	6.7	4.2	3.4	7.9	8.0	8.8	2.1	6.8	7.7
	0.055		0.824		0.999		0.996		0.206	

Mono, monosynaptic; poly, polysynaptic;); (f)AHP, (fast) after‐hyperpolarizing potential; RMP, resting membrane potential; dec time, decay time; *τ*_m_, membrane time constant; R_m_, input resistance; C_m_, capacitance; T, tonic; D, delayed; P, phasic; H, H‐current; TP, tonic‐phasic. Green boxes highlight statistically significant differences (*P* > 0.05).

The kinetics of the EPSC can provide clues about synapse location on the dendritic tree (Magee 2000; Marlin and Carter 2014; Straub et al. 2016). So, we next compared EPSC kinetics among the five cell types (Fig. 3D). Characterization of monosynaptic TRPV1/ChR2 EPSCs showed that the latency to EPSC onset was markedly longer in synapses with tonic neurons (Fig. 3E). To our surprise, the EPSC kinetics also differed by cell type (Fig. 3F–G). For example, EPSCs in tonic cells had markedly slower kinetics as evidenced by the prolonged rise time and decay time constant, while delayed cells had a notably smaller decay time constant. We next investigated minimal light‐evoked EPSC amplitudes (Fig. 3 H) and found these to differ considerably across cell types. Tonic neurons had the least variability in minimal EPSCs and had consistently small EPSC amplitudes, while delayed neurons showed great variability in minimal EPSC amplitudes ranging from 20 to 220 pA.

We then compared electrophysiological properties of cells that received mono‐ versus polysynaptic input (Table 2). Tonic neurons with monosynaptic inputs had a higher rheobase and an increased latency compared to those with polysynaptic input. These neurons also had a higher capacitance and decreased input resistance. Similarly, phasic neurons with monosynaptic input had a much longer latency to spike, and trended toward a higher rheobase. Properties of delayed neurons did not differ greatly depending on the type of input. However, it is interesting to note that delayed neurons with monosynaptic input had a trend toward cell body locations at a greater distance from the trigeminal tract, whereas delayed cells with polysynaptic input trended to be closer to the tract.

### Morphology of superficial laminae neurons segregated by cell types

**Figure 4:**
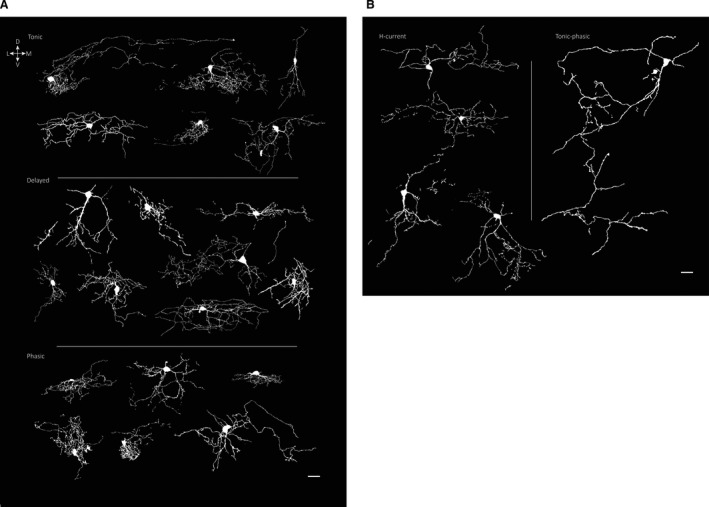
Morphology of laminae I/II TNc neurons separated by cell type. Reconstructions of confocal images of biocytin‐labeled neurons. (A) Morphology of tonic (top), delayed (middle), and phasic (bottom) neurons. (B) Morphology of H‐current (left) and TP (right) cells. The cells are oriented relative to the dorsal surface as indicated. Scale bar, 20 *μ*m.

A subset of TNc neurons were intracellularly labeled with biocytin for post hoc analysis using fluorescent staining and confocal microscopy to see whether cells in different electrophysiological classes also fell into anatomical groups (Fig. 4 and Table 3). Examples of biocytin‐labeled neurons are shown in Figure 4, grouped according to their firing pattern. Tonic and phasic neurons had relatively small somata, and usually fine bushy dendritic arbors. Delayed cells tended to have larger cell bodies and were less ramified. H‐current cells had larger diameter, less ramifying dendritic trees. While we only recovered a single TP cell, this neuron had large and extensive dendrites perpendicular to the superficial border of the nucleus.

**Table 3:** Morphometric parameters of reconstructed neurons. Data represent mean ± S.E.M.; DV, dorso‐ventral extent, ML, medio‐lateral extent in coronal (transverse) slices. Green boxes highlight statistically significant differences (p > 0.05)

Cell type	*n*	DV (mm)	ML (mm)	Soma (mm)
Tonic	6	80.31 ± 13.24	170.6 ± 40.10	7.25 ± 0.37
Delayed	8	101.4 ± 7.66	134.7 ± 18.59	8.82 ± 0.59
Phasic	6	83.41 ± 12.66	109.1 ± 26.46	7.61 ± 0.42
H‐current	4	147.7 ± 23.88	185.7 ± 24.67	8.70 ± 0.88

### Properties of TNc reporter neurons

**Figure 5:**
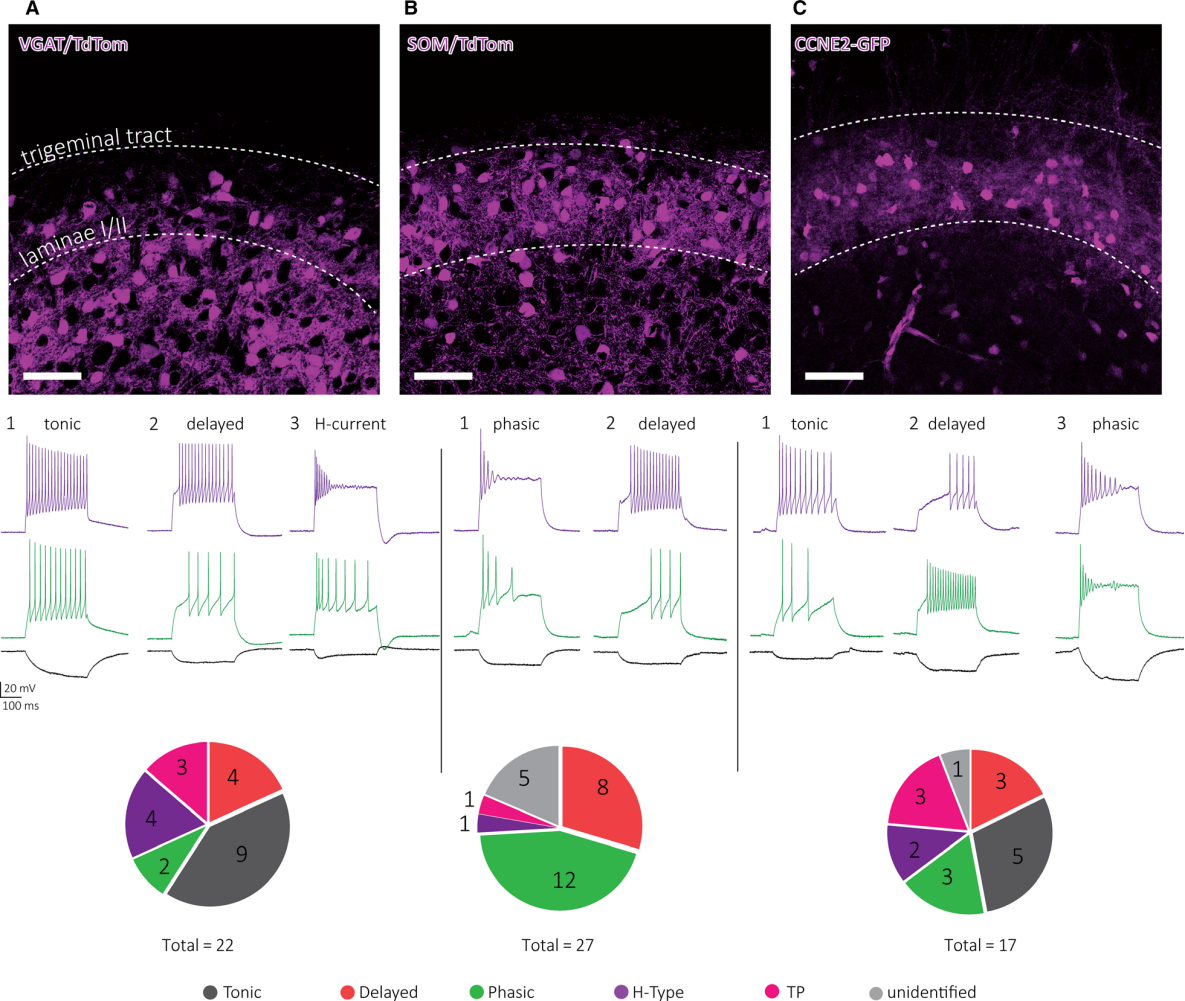
Properties of genetically labeled TNc neurons. Top, representative confocal images of VGAT/TdTom neurons (A), SOM/TdTom neurons (B), and CCNE‐GFP neurons (C) in the TNc. White‐dotted lines indicate border to trigeminal tract (top) or border between laminae II and III (bottom). Scale bar indicates 50 *μ*m. Middle, Representative voltage traces of most frequent firing patterns are illustrated for each genetic reporter line. Colors of voltage traces indicate different amount of current injected (purple: +200 pA; green: +100 pA; black: ‐25 pA). Bottom, pie charts illustrate the frequency of each cell type from each genetic reporter line.

Next, we used fluorescent reporter mouse lines to characterize the properties of genetically identified cell populations. VGAT‐lineage cells comprise all inhibitory interneurons, labeling cells that express the vesicular transporter for GABA and glycine. SOM‐lineage neurons express the neuropeptide somatostatin, and in the spinal cord are comprised of predominantly excitatory neurons (Yasaka et al. 2010; Duan et al. 2014). CCNE2‐lineage cells transcribe cyclin‐E2, which is a regulator of CDK kinases and is localized in superficial laminae of the TNc; our data indicate that cells that express CCNE2 do not seem to be enriched in any of our classification groups. VGAT‐lineage cells were mostly found in deep TNc laminae, but could also be seen in superficial lamina II and occasionally lamina I (Fig. 5A, top). The electrophysiological properties of VGAT neurons varied considerably (Fig. 5A, middle and bottom). Tonic neurons were the most prominent group (9/22); however, delayed (4/22) and H‐type (4/22) cells were also found frequently. SOM‐lineage cells were strongly represented in the superficial laminae (Fig. 5B, top) and primarily comprise phasic neurons (12/27) and a large proportion of delayed cells (8/27) (Fig. 5B). CCNE2 neurons were mostly found in superficial laminae I/II (Fig. 5C, top). Immunostaining revealed that these neurons co‐label with vesicular glutamate transporter 2 but also glutamic acid decarboxylase 65/67, markers for excitatory and inhibitory interneurons, respectively (Bruno Pradier, Julie Kauer unpublished observation). All of the five cell types defined in this study were present among CCNE2 lineage neurons with a frequency ranging from 10% to 30%, among which tonic neurons were the most frequent cell type; thus, this marker may not usefully distinguish cell groups in the TNc.

### Unsupervised clustering analyses

**Figure 6:**
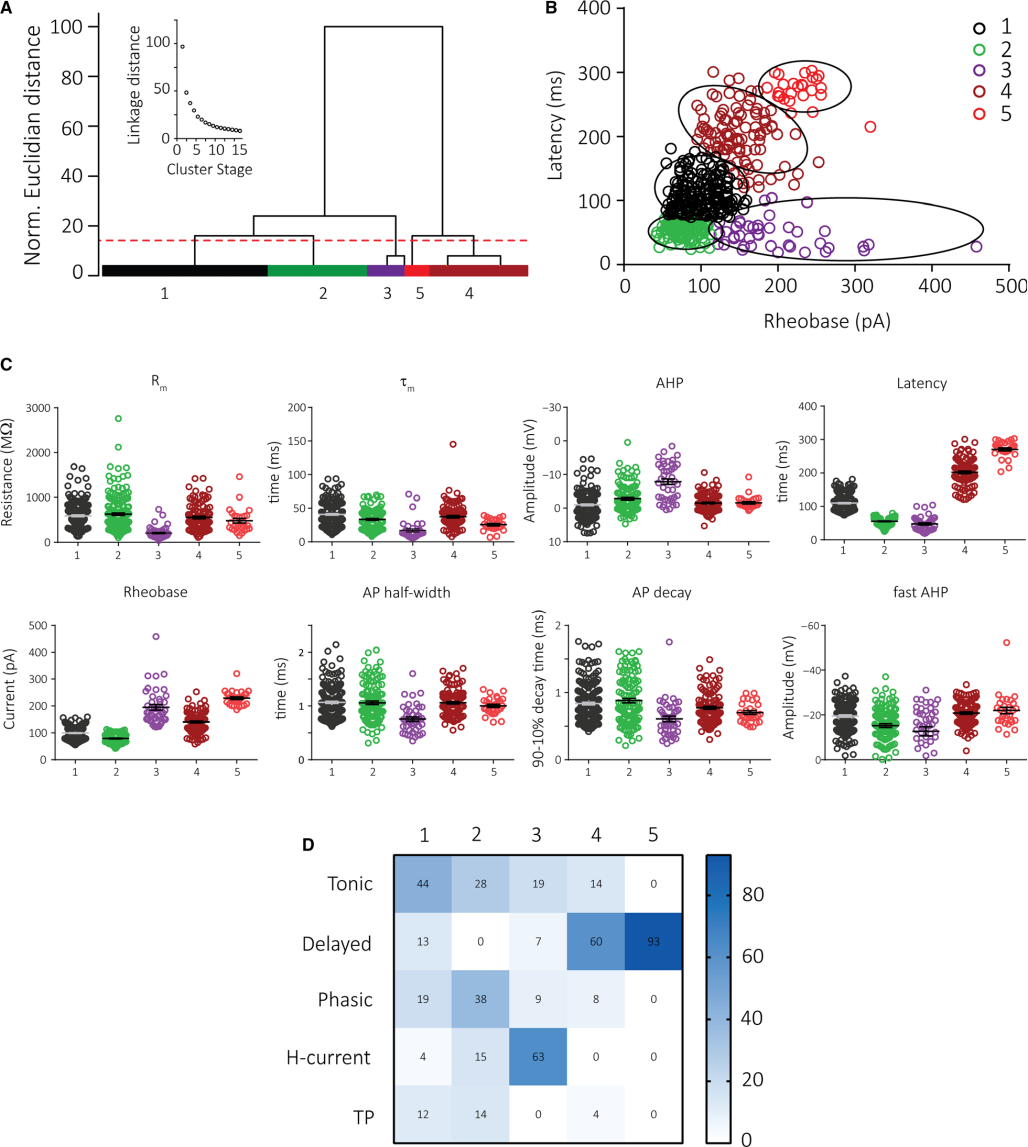
Ward's clustering of intrinsic properties of TNc neurons identifies five subgroups of neurons that correlate well with previously described cell types. (A) Dendrogram of Ward's unsupervised cluster analysis of 491 patched neurons using eight parameters. The *x*‐axis displays single neurons and *y*‐axis indicates Euclidian linkage distance with larger linkage distance indicating larger dissimilarity. Cutoff (dotted line) identifies five subgroups. (B) X‐Y scatter plot of 491 neurons displaying latency (y) and rheobase (x) shows distinct separation of clusters 1‐5. Single clusters are highlighted with black ellipses. (C) Representative electrophysiological properties by Ward's cluster type. (D) Percentage frequency of manually classified cell types found in each cluster. Each cluster has one predominant cell type; 1‐3 are mainly tonic, phasic or H‐current cells, respectively, whereas clusters 4 and 5 primarily contain delayed cells.

Next, we explored avenues to functionally segregate cell types based on their physiological properties in an unsupervised cluster analysis. We first aimed at replicating our previously described cell types and performed a cluster analysis including eight parameters (amplitude, half‐width, rheobase, latency, fAHP, AHP, rise, and decay time) using the Ward's method (Ward 1963). With this analysis, we identified two main groups of neurons that were functionally different with one branch containing primarily delayed neurons (Fig. 6A). Distribution of the properties of these clusters showed that rheobase and latency are the most important predictors separating cell groups (Fig. 6B, C and S1). We used a cutoff of 15% of the maximal Euclidian distance, which resulted in two main groups that gave rise to five final clusters (inset Fig. 6A). Overall, these clusters correlate well with our previously defined neuronal classes: clusters 1 (*n* = 191), 2 (*n* = 117), and 3 (*n* = 43) were functionally close and primarily comprised tonic, phasic, and H‐current cells, respectively (Fig. 6D). TP cells did not clearly separate into one single group, but were rather found in clusters 1 and 2 (Fig. 6D). Delayed neurons mainly produced two isolated clusters (4 and 5), which were functionally close. Neurons in cluster 4 (*n* = 112) had a shorter latency and markedly lower rheobase compared to cells in cluster 5 (*n* = 28) indicating that subtypes might exist within delayed neurons.

**Figure 7:**
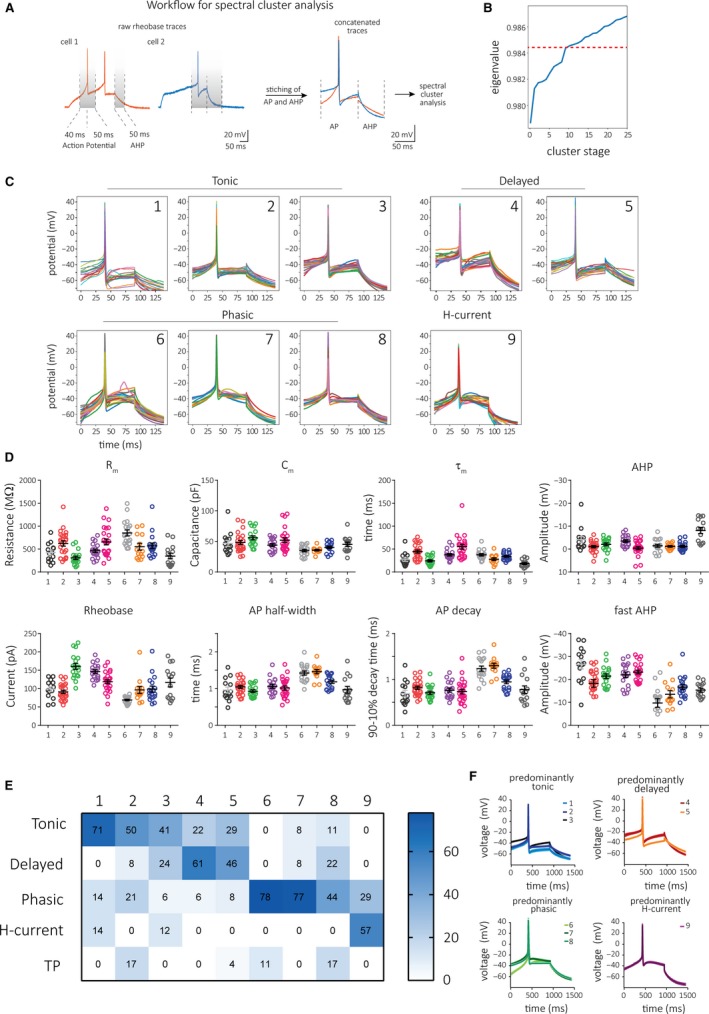
Spectral clustering of concatenated traces of rheobase APs and AHPs is sufficient to segregate TNc neurons into functionally different groups. (A) Workflow of waveform processing used for unsupervised spectral clustering analysis of 161 neurons. Waveforms were cut as indicated by the dotted lines and then lined up on the *x*‐axis at the AP peak; 40 ms prior and 50 ms following the peak were concatenated with an additional 50 ms of the AHP. (B) Plotting eigenvalues as function of cluster stage indicates the decreasing dissimilarity between clusters with progressing cluster stage. Cutoff (dotted line) used here to identify nine subgroups. (C) Graphs of individual voltage traces from all cells in nine clusters. (D) Electrophysiological properties of cells in each clusters. (E) Percentage frequency of previously defined cell types found in each cluster. Each cluster includes one predominant cell type. (F) Averaged AP/AHP traces (± S.E.M.) for predominantly tonic, delayed, phasic, and H‐current cells containing clusters show distinctive features of each cluster.

**Figure 8:**
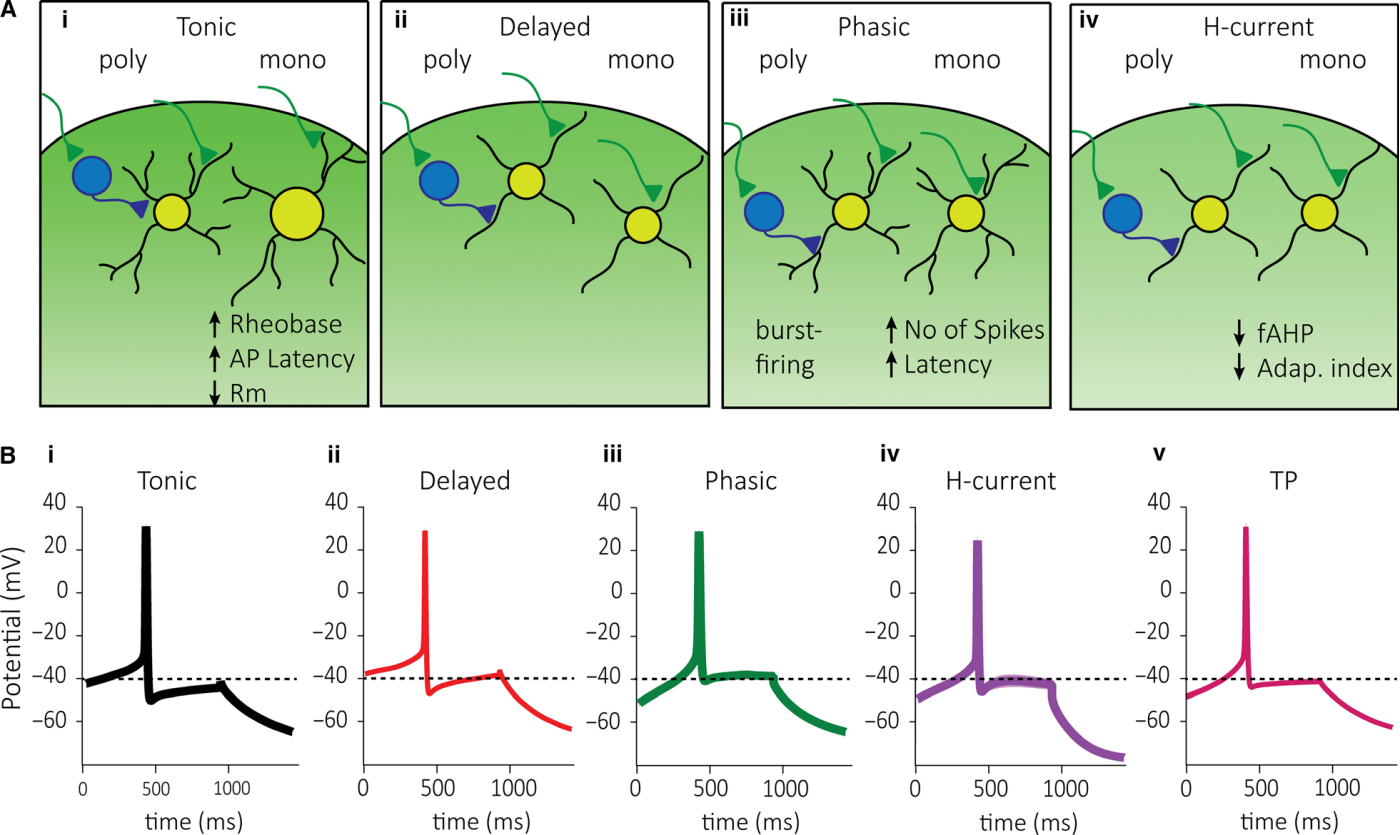
Graphic summary of neurons receiving poly‐ versus monosynaptic inputs segregated by cell type. (Ai) Tonic cells receiving monosynaptic input may form more distal synapses with TRPV1/ChR2 afferents. Moreover, these cells also had a lower R_m_, and increased latency to AP and rheobase current compared to tonic cells receiving polysynaptic input. (Aii) Delayed neurons with monosynaptic input may have more proximal synapses with TRPV1/ChR2 afferents and more likely to be present in lamina II than lamina I. (Aiii) Phasic neurons may receive more proximal monosynaptic TRPV1/ChR2 input and had increase latency to spike and increased number of spikes. Phasic neurons with polysynaptic innervation showed most frequently burst firing. (Aiv) H‐current cells receive a faster and perhaps more proximal monosynaptic TRPV1/ChR2 input and had a lower fAHP with stronger adaptation of AP firing. (B) Averaged AP shape of cells classified as tonic (i), delayed (ii), phasic (iii), H‐current (iv), and TP (v) neurons. It should be noted that while we refer to this type of input as “polysynaptic,” similar synaptic current shapes might instead be generated by asynchronous release (Peters et al. 2010).

We observed a qualitative heterogeneity of cell firing pattern, which at times made classification difficult across the five cell types. However, the shape of rheobase action potentials appeared to distinguish cell groups, and we therefore performed an unsupervised spectral clustering analysis of spike shapes (see Fig. 1B and 8B). For this analysis, we picked three portions of the action potential waveform. We included the periods 40 msec prior and 50 msec after the action potential peak; since the AHP also seemed strikingly different from cell to cell, we also added a 50 msec period starting at the end of the depolarizing step. Together, this created a concatenated trace of 140 msec which we will refer to as the rheobase action potential (Fig. 7A). We hoped that this portion of the data for each cell would include not only easily quantifiable properties (such as were measured for Fig. 6), but other membrane potential changes around the spike and repolarization that likely depend on complex voltage‐dependent conductances that could in theory vary by cell class. The steep increase of eigenvalues (obtained by the spectral clustering) during the first cluster stages indicates large dissimilarity between clusters, whereas smaller changes in eigenvalues occurring with later cluster stages indicated increased similarity between clusters. Since choosing a higher cutoff leads to a larger number of clusters that would probably lack meaningful separation of cells, we defined the cutoff following the final large increase in eigenvalues, which was observed from stage 8 to 9, resulting in nine clusters (dotted line Fig. 7B). Individual traces of rheobase action potentials for each cluster are shown in Figure 7C 1‐9. This method produced a surprisingly good segregation of spike shapes not only based on spike kinetics or AHPs, but also on dV/dt prior to the spike threshold and following the fAHP. We also noted that not only the active properties of the cell (values we had used as input for the analysis), but even the passive properties (such as R_m_ and *τ*_m_) differed among the nine groups (Fig. 7D and Fig. S2). We then investigated the extent to which each cluster included the previously described cell types using the manual classification (Fig. 7E). Clusters 1–3 (1: *n* = 14; 2: *n* = 24; 3: *n* = 18) were predominantly comprised of tonic neurons, clusters 4–5 (4: *n* = 18; 5: *n* = 24) contained mostly delayed firing cells, while clusters 6–8 (6: *n* = 18; 7: *n* = 13; 8: *n* = 18) comprised mainly phasic neurons and cluster 9 (*n* = 14) of H‐current cells. Averaging individual rheobase spike traces accentuates similarities within and dissimilarities between predominantly tonic, delayed, phasic, and H‐current cell clusters (Fig. 7F). Taken together, our results suggest a functional segregation of tonic, delayed, phasic, and H‐current cells and that these cells show further physiological differences depending on TRPV1/ChR2 inputs (Fig. 8A). Further, we demonstrate that a functional segregation of cells is also possible based on the shape of rheobase spikes (Fig. 8B).

## Discussion

### Neurophysiological properties of different TNc cell types

We have analyzed and classified the firing pattern of 535 neurons in the superficial laminae of the TNc. Most of the neurons of the superficial laminae of the dorsal horn and its extension into the brainstem TNc are interneurons. Considerable earlier work has been done in the dorsal horn to classify these interneurons, based on morphology, identifying vertical, islet, central, and radial cells (Grudt and Perl 2002; Yasaka et al. 2010). In our analysis, we relied on previously described methods to classify neurons based on physiological firing patterns, and identified tonic, phasic, and delayed cells (Yoshimura and Jessell 1989; Lopez‐Garcia and King 1994; Grudt and Perl 2002; Sedlacek et al. 2007; Davies and North 2009; Alba‐Delgado et al. 2015). However, due to their unique physiological properties and observed frequency in the TNc, we also propose two additional groups, H‐current cells and TP cells.

#### H‐current cells

We propose a new electrophysiological category for TNc neurons; H‐current cells can not only be identified by their sag‐potential (or sag‐current), but also by their fast membrane time constant, fast AP kinetics, and large AHP, with the two latter properties being sufficient to classify them as a separate group in unsupervised clustering analyses.

While the presence of I_h_ in TNc and spinal cord neurons has been observed previously (Yoshimura and Jessell 1989; Lopez‐Garcia and King 1994; Grudt and Perl 2002; Melnick 2008; Yasaka et al. 2010; Alba‐Delgado et al. 2015; Dougherty and Chen 2016), H‐current cells were not described as a separate group. I_h_ currents are temperature‐sensitive (DeCoursey and Cherny 1998; Fujiwara et al. 2012) and some cells may have been overlooked due to smaller currents in studies that were recorded at RT (Grudt and Perl 2002; Yasaka et al. 2010), whereas in our TNc neuron recordings at warm temperatures, we observed currents up to 320 pA. In the spinal dorsal horn, these cells have been most frequently found grouped as tonic firing islet cells (Melnick 2008), and also in some lamina I projection neurons with high I_h_ (160pA) (Grudt and Perl 2002). Yasaka et al. (2010) reported that cells expressing sag‐current co‐label with biomarkers for inhibitory (VGAT), but not excitatory neurons (VGLUT2). In line with this, we found H‐current to be present in a subset (4/22) of inhibitory VGAT/TdTom‐cells. I_h_ currents often lead to rebound spiking following hyperpolarization, a property that has been associated with pace‐making activity (Luthi and McCormick 1998). Accordingly, H‐current expressing cells exhibited the highest rate of spontaneous firing in an in vitro spinal cord‐leg preparation (Lopez‐Garcia and King 1994), and the spontaneous firing was halted during cutaneous stimulation, suggesting that these cells receive strong inhibitory input. In our study, direct activation by (excitatory) primary afferents was noted, but our experiments were carried out in picrotoxin, preventing our ability to note feed‐forward inhibition that may normally be present in these cells. Recently, I_h_ currents were reported to be present in more than half of the neurons in the superficial laminae of rat TNc (Alba‐Delgado et al. 2015), including PKC*γ* cells thought to label excitatory neurons in the spinal dorsal horn (Polgar et al. 1999). However, in contrast to our population of H‐current cells these neurons in rat TNc had a low threshold and on average a moderate I_h_ of ~20 pA, a value comparable with values observed in many tonic and delayed neurons found in our study (we only included cells as H‐current cells if the sag‐current was > 25pA). As we describe H‐current cells with an average of 140pA, we propose to discriminate cells with low/moderate and high I_h_. In the rat dorsal horn, large I_h_ currents (>40pA) were noted in a quarter of inhibitory lamina II neurons but no excitatory neurons (Yasaka et al. 2010), and they were strongly associated with the islet cell morphology (Melnick 2008). In a single synaptically connected cell pair (H‐current cell synapsing onto another H‐current cell), we found that the presynaptic neuron released both GABA and glycine at synapses on the postsynaptic H‐current neuron (Kelsey Barcomb, Bruno Pradier, Julie Kauer, unpublished observations), further suggesting that at least some of the H‐current cells are inhibitory.

An additional property of the cells we classed as H‐current cells in the TNc is the presence of a marked AHP (peaking within 20–160 msec) following a depolarizing step, also noted previously in spinal cord islet cells (Melnick 2008). AHPs generally result from distinct underlying K^+^ currents, and although the AHP and I_h_ sag are functionally independent, they were highly correlated in this cell type. In two cells in which ZD7288 was applied, both the sag‐current and the AHP were blocked. We speculate that the HCN channels that underlie the I_h_ in these neurons allow permeation of Ca^2+^ (Michels et al. 2008), and may be closely associated with Ca^2+^‐activated K^+^ channels that could underlie the AHP.

#### TP cells

Along with tonic‐ and phasic firing cells, we frequently observed cells that would exhibit both firing patterns. With greater depolarizing steps, these cells switched to a phasic firing pattern, perhaps due to depolarization block of Na^+^ channels (Tucker et al. 2012). Since these cells made up approximately 10% of all recorded cells, we grouped them as TP cells. However, these cells have not been noted previously in the TNc or perhaps have been grouped as either tonic or phasic neurons; the electrophysiological properties of TP cells either resemble tonic or phasic neurons thereby suggesting that TP cells may represent an intermediate form. Consistent with this idea, we found TP cells to be assigned either to predominantly phasic or tonic cell clusters in both unsupervised cluster analyses. Since cell types exist in a continuum rather than distinct groups, tonic or phasic neurons might change firing properties and convert to one another; however, this balance may also be tipped under different modulatory physiologic and pathologic conditions. In line with this idea, blockade of I_M_ K^+^ currents have been demonstrated to convert phasic‐ into tonic firing cells (Wang et al. 1998). Further supporting this general idea, we and others have observed that upon blockade of I_A_ currents in delayed cells these cells switch from delayed to fast‐onset spiking (Yoshimura and Jessell 1989). More recently, it was also shown that peripheral capsaicin injection decreased I_A_ currents in somatostatin‐containing cells, which then switched from delayed to fast‐onset tonic firing cells (Zhang et al. 2018).

#### Tonic, phasic, and delayed cells

Although overall, H‐current cells were the most distinct group across multiple cell properties (Fig. 2), we also noted distinct properties for the other groups as well. For example, phasic cells had a lower capacitance and higher R_m_ than tonic or delayed cells; phasic cells also had a longer duration action potential, with a slow falling phase, and a small fAHP. Neurons with phasic firing properties were noted in a rat dorsal horn study to be nociceptive specific, activated only by high‐intensity peripheral stimuli (Lopez‐Garcia and King 1994). Dorsal horn neurons genetically labeled for vGluT2, and therefore presumably excitatory, were associated with longer duration APs than either GAD67‐ or GlyT2‐labeled presumptive inhibitory cells (Punnakkal et al. 2014). However, these cells also had a significantly lower rheobase, in contrast to our findings of higher average rheobase in phasic cells. It is worth noting that the discrepancy in absolute number might arise from the use of different protocols to determine the rheobase (current steps vs. ramp).

Tonic cells were our most prevalent cell class, and this firing pattern has previously been observed in inhibitory neurons (Punnakkal et al. 2014). Consistent with our results, GAD67‐EGFP‐labeled cells had higher membrane capacitance and larger APs than nonlabeled cell types, again suggesting their likely inhibitory identity (Punnakkal et al. 2014). Moreover, this spiking pattern is consistent with Group A neurons (Lopez‐Garcia and King 1994), a cell group characterized in adult rats as wide‐dynamic range cells responsive to both low‐ and high‐threshold sensory afferent input.

Delayed cells exhibited a higher rheobase, shorter AP, as well as identifying longer latency to first spike. Our visual classification of cells into tonic, phasic, and delayed groups exhibited similar proportions to those previously characterized in juvenile rat lamina I of TNc (Sedlacek et al. 2007) and mouse lamina II (Davies and North 2009), although another study in rat reported no delayed cells at all (Alba‐Delgado et al. 2015). In accord with the rat study (Sedlacek et al. 2007), we found that delayed cells have a greater rheobase compared to tonic cells, and delayed cells have a broader AP than either tonic or phasic cells. In contrast, we did not observe a large effect size comparing the RMP and R_m_ in the three cell types, although others found that RMP was more depolarized in tonic cells than in either delayed or phasic cells both in mice and in rats (Sedlacek et al. 2007; Davies and North 2009). As has been noted previously (Yasaka et al. 2010), the use of only a single holding potential can blur distinctions among cell classes, and this may account for some of the differences we observed. Nonetheless, the large number of neurons in our sample, as well as the clear separations between cell classes using either the Ward's method or the spectral cluster analysis, provides some confidence that major distinctions can still be observed.

Notably sparse among our cell types were single‐spiking cells, frequently reported in dorsal horn neurons (Jo et al. 1998; Ruscheweyh and Sandkühler 2002), and also reported previously in the TNc of rat and mouse (Sedlacek et al. 2007; Alba‐Delgado et al. 2015). Our sample size is far greater than in previous TNc reports, so it is unlikely that we missed this population; indeed in our unrelated work recording in the lumbar dorsal horn slice, we encounter single‐spiking neurons frequently (Kloc, Pradier and Kauer, unpublished). Instead, our data indicate that single‐spiking neurons are much less frequent in the mouse TNc at age p14‐35 and recorded at 28°C than in the dorsal horn preparation. Recent work suggests that in neurons expressing gastrin‐releasing peptide, the single‐spiking phenotype is seen in a third of cells (Dickie et al. 2019); it is possible that neurons expressing this peptide more sparse or absent in the TNc.

### Reporter neurons

We found tonic cells to be the most frequently encountered cell type in the TNc. This firing pattern was seen in about 40% of inhibitory VGAT/TdTom cells but in none of SOM/TdTom cells, which in the spinal cord instead comprises a population of rather excitatory neurons (Yasaka et al. 2010; Duan et al. 2014). Consistent with this observation, in the spinal dorsal horn, the majority of tonic cells were identified as GABA‐ or glycinergic interneurons (Heinke et al. 2004; Yasaka et al. 2010; Punnakkal et al. 2014). However, this cell type might also be infrequently found among excitatory interneurons, including PKC*γ*‐reporter neurons in the rat TNc (Yasaka et al. 2010; Alba‐Delgado et al. 2015).

Delayed cells were the second most frequently encountered cell population in the TNc, characterized by the long delay of action potentials, likely caused by the high density of repolarizing I_A_ current (Pradier and Kauer, unpublished observations; Yoshimura and Jessell 1989; Ruscheweyh et al. 2004). This firing pattern is most commonly associated with spinal cord excitatory neurons (Yasaka et al. 2010; Punnakkal et al. 2014), and we consistently found SOM/TdTom neurons exhibiting this firing pattern. However, we also found delayed cells among inhibitory VGAT/TdTom neurons, hinting at possible region‐specific differences in cell distribution. However, VGAT/TdTom‐delayed cells typically had relatively short delays, suggesting a reduced I_A_ current density.

### Synaptic TRPV1/ChR2 inputs

Virtually, every neuron in lamina I/II of the TNc received input from TRPV1/ChR2 fibers. Interestingly we found that EPSCs that appeared to have multiple components (that we refer to as polysynaptic) occurred most frequently in delayed firing cells and were more likely in lamina I rather than in lamina II neurons. This suggests strong feed‐forward excitation and probably recurrent activity under conditions of disinhibition (i.e., our recording condition) especially for delayed cells. On the other hand, H‐current cells and large tonic cells were more likely to receive monosynaptic TRPV1/ChR2 input. However, it remains possible that EPSCs with multiple peaks may result from asynchronous release seen at many synapses including the brainstem (Peters et al. 2010). If so, our data would indicate that delayed cells in lamina I receive inputs characterized by asynchronous release. In four cells in a previous study (Pradier et al. 2018), however, the polysynaptic component was blocked in TRPV1/ChR2 afferents after treatment with TTX and 4AP. Multiple EPSC components caused by asynchronous release should not be blocked under these conditions.

While the use of optogenetically identified inputs allows us to selectively activate subsets of peripheral TRPV1‐cre afferents, it does not allow discriminating C‐ from A*δ*‐fibers, or nociceptors from low‐threshold mechanoreceptors (LTMR,) all of which (with varying frequencies) express ChR2 (Pradier et al. 2018). To investigate this potential synaptic input diversity, we were interested whether these inputs might have different properties. We therefore measured kinetics of monosynaptic EPSCs (latency to onset, rise time, and *τ* decay) to investigate electrophysiological differences of inputs across cell types. To our surprise, we found a striking increase in latency to onset for monosynaptic EPSCs in tonic cells. Classically, latency is used to discriminate C from A*δ*‐fiber inputs based on their different conduction velocities (Ringkamp et al. 2013). Due to the strongly reduced afferent length in our slice preparation and the optogenetic dynamics potentially allowing axonal depolarization close to the terminals, this measure is more complex to interpret. However, when we looked at the EPSC kinetics, it was striking again that the EPSC shape differed by cell type, with tonic cells displaying the slowest rise and decay kinetics. EPSC shape can inform about the location of the synapse relative to the soma with distal synapses showing a slower kinetic compared to proximal synapses (Magee 2000; Marlin and Carter 2014; Straub et al. 2016), although there are multiple parameters that may influence this measure. Further, we found minimal EPSCs and the number of spikes evoked at maximal stimulation to be the smallest in tonic neurons. Thus, the slower EPSC kinetics, smaller minimal EPSC amplitude, and increased latency to EPSC onset suggest that tonic neurons receive more distal TRPV1 input compared to other cell types. Increased dendritic filtering in tonic cells would be consistent with the more complex morphology of dendrites in these neurons. This is surprising since despite the relatively small and slow EPSCs generated with synaptic activation, tonic cells were relatively excitable in response to direct current injection into the soma. Other cell types may have more proximal synapses with TRPV1‐fibers or less dendritic filtering modifying EPSC shape. It is also worth noting that inputs of H‐current cells often followed up to 20 Hz stimulation indicating that these cells are less likely to be innervated by TRPV1‐C‐ or A*δ*‐fibers, but rather by TRPV1‐LTMRs (Pradier and Kauer, unpublished observation). As noted above, these cells may also receive strong inhibitory feed‐forward inhibition not observable under our recording conditions (Lopez‐Garcia and King 1994).

### Morphology

Our qualitative study of neuronal morphology included cell fills of 25 neurons allowed us an overview of whether different physiological cell types share similarities, although we only morphologically characterized a small subset of cells recorded from this study. We found that both tonic and phasic cells have a bushy dendritic tree. Since our recordings and staining were only done using transverse slices, we can only speculate that these cell types might correlate with islet or central cells characterized in parasagittal slices in the spinal cord (Grudt and Perl 2002; Yasaka et al. 2010). Delayed cells in TNc had more multipolar morphology as described earlier (Sedlacek et al. 2007), and with fewer dendritic ramifications than tonic and phasic cells.

### Unsupervised classification

For the supervised classification, we explicitly used fewer categories to reduce potential bias. However, since this method is inherently biased based on experimenter‐determined input criteria and certain neuronal properties or firing patterns may also change depending on experimental conditions (i.e., magnitude of depolarizing current, recording temperature, blockade of GABA_A_, and glycine receptors, but also physiological or pathological state of animals used), we next explored statistics‐based approaches that would allow us to (1) replicate our supervised clustering results (independently of information on AP firing) and (2) identify further neuronal subsets. To address the first question, we used Ward's clustering algorithm as previously described (Leist et al. 2016; Ferrante et al. 2017; Abrahao and Lovinger 2018) with eight parameters and found that latency and rheobase currents were surprisingly important predictors to segregate delayed cells from tonic, phasic, and H‐current cells. Further, we were able to separate a predominantly H‐current cell cluster from tonic and phasic neurons based on rheobase, AP kinetics, and AHP. The remaining two clusters showed the most heterogeneity with each containing approximately 40% tonic and phasic cells, respectively, and that differed most prominently in rheobase, latency, AHP, and capacitance. This outcome was very surprising since none of these parameters (with the exception of AHP) were relevant for the supervised classification and shows a strong correlation of these properties with the respective firing pattern. While we found this method to predict the supervised classification with good success, we also realized that some features of AP shapes were not captured by our analysis (e.g., dV/dt before AP threshold and after fAHP) that might be important for defining subgroups (see Fig. 8B). We hypothesized that including the spike shape as a whole together with the AHP might be used as a further predictor for cell clusters. We therefore used the spectral cluster analysis, which compares the similarity of trace shapes. This method produced nine clusters and identified three predominantly phasic, three tonic, two delayed, and one H‐current cell cluster. These clusters differed significantly in their spike shape, but also in their intrinsic properties, which potentially identifies new subgroups within the larger groups of tonic, delayed, and phasic cells. Intriguingly, we found that this method could cluster cell groups with properties that are not directly reflected in the voltage samples we provided the model. For example, clusters 8 and 9 have strikingly different resting membrane potential, capacitance, and rheobase, while clusters 6 and 7 differ sharply in membrane time constant. The spectral clustering algorithm therefore may be a useful method for grouping functionally different cell types based on short stretches of data around the AP for future studies.

## Conflict of Interest

The authors declare no conflicts of interest.

## Supporting information




**Figure S1**. Electrophysiological properties of lamina I/II neurons in the TNc segregated into five groups by Ward's method.

**Figure S2**. Electrophysiological properties of lamina I/II neurons in the TNc segregated into nine groups by the spectral clustering algorithm.

**Table S1**. Electrophysiological properties of TNc reporter neurons. In each row, averages are represented (top) followed by S.E.M. AP, action potential; (f)AHP, (fast) after‐hyperpolarizing potential; RMP, resting membrane potential; τ_m_, membrane time constant; R_m_, input resistance; C_m_, capacitance.
