# Supplementary figures and images for: Properties of neurons in the superficial laminae of trigeminal nucleus caudalis
Source: Physiol Rep. 2019 Jun 18;7(12):e14112. doi: 10.14814/phy2.14112 (PMC6581829; doi:10.14814/phy2.14112)

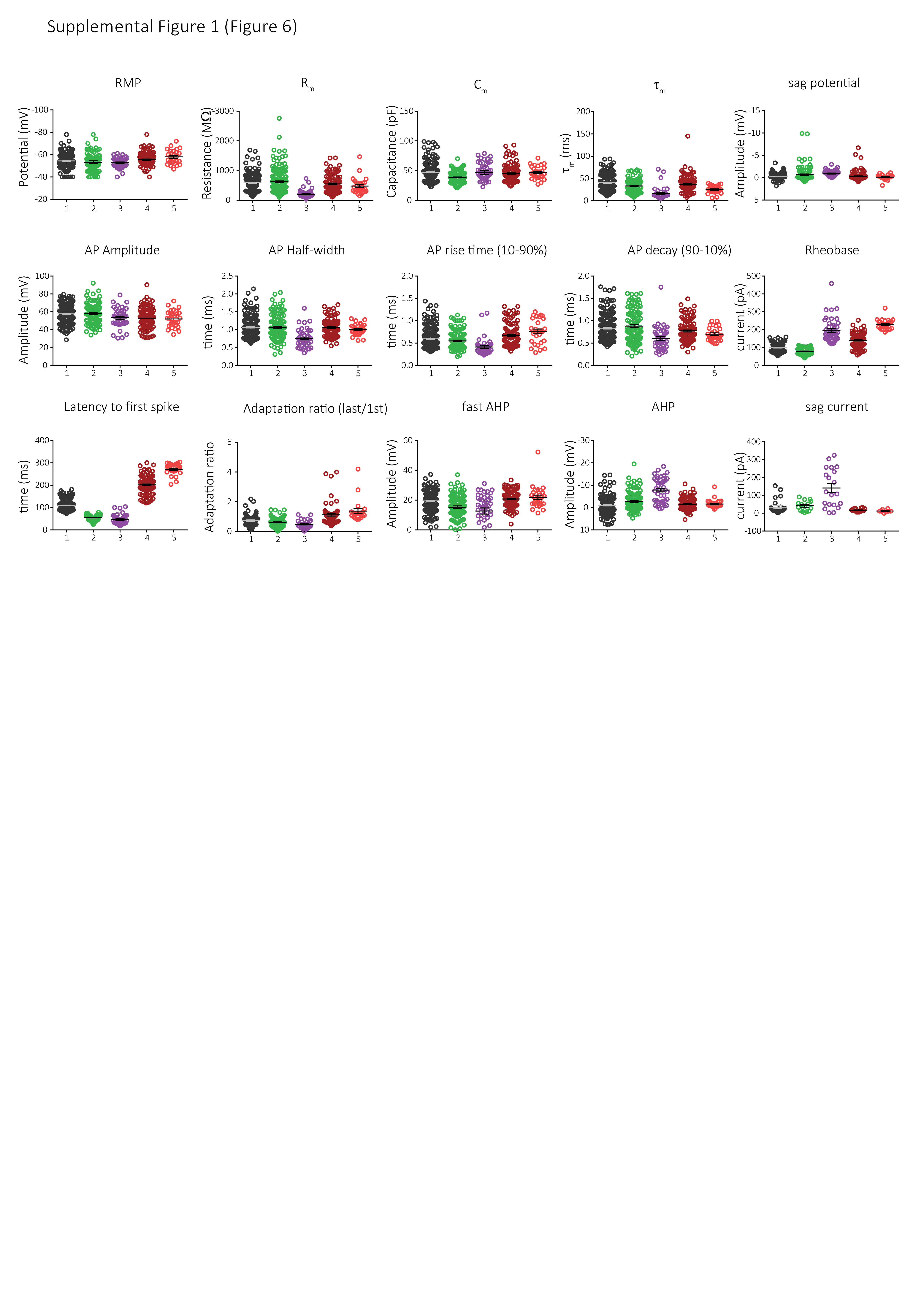

Supplement: Supplementary file 1 — Figure S1. Electrophysiological properties of lamina I/II neurons in the TNc segregated into five groups by Ward's method. [file PHY2-7-e14112-s001.png]

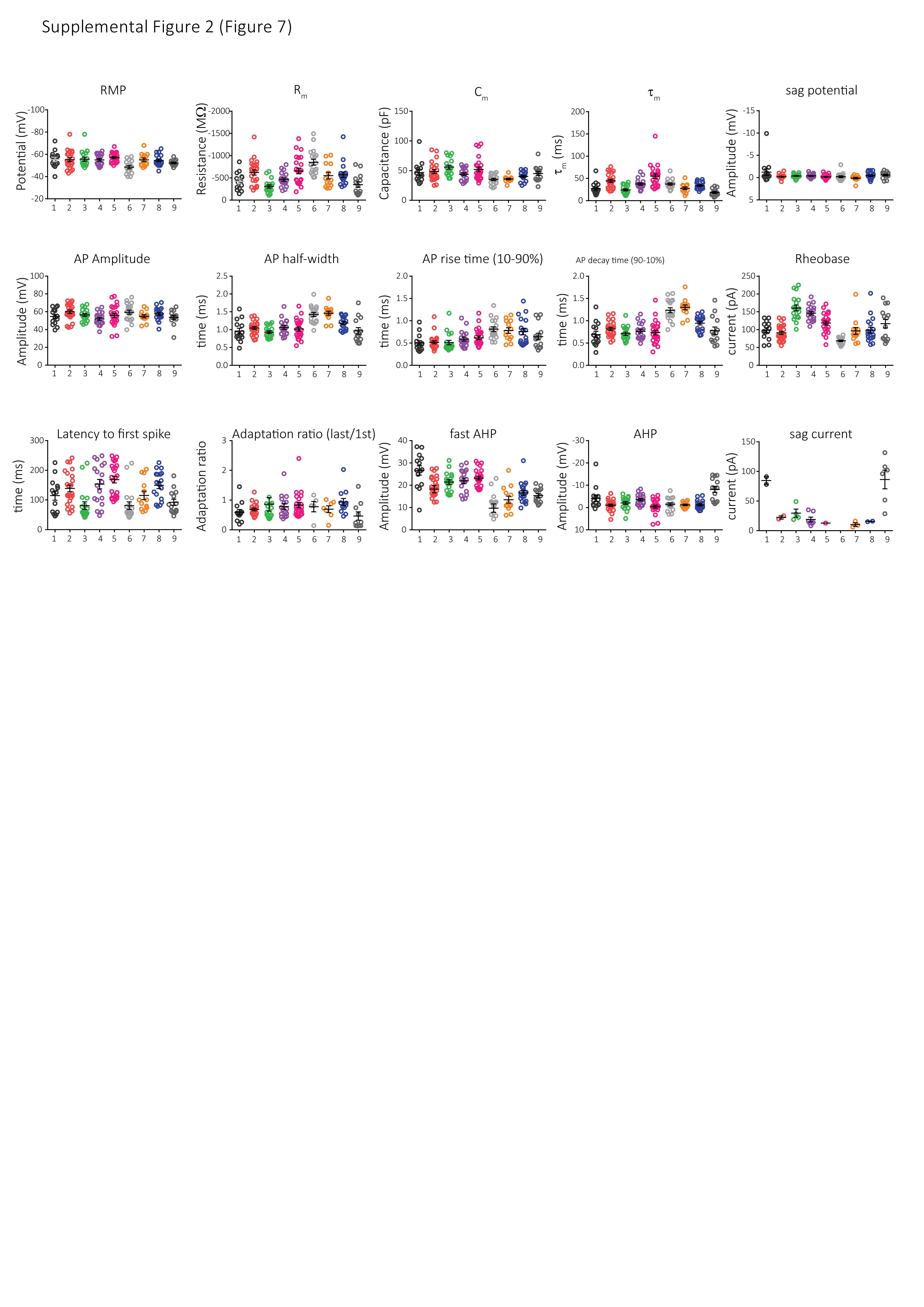

Supplement: Supplementary file 2 — Figure S2. Electrophysiological properties of lamina I/II neurons in the TNc segregated into nine groups by the spectral clustering algorithm. [file PHY2-7-e14112-s002.png]

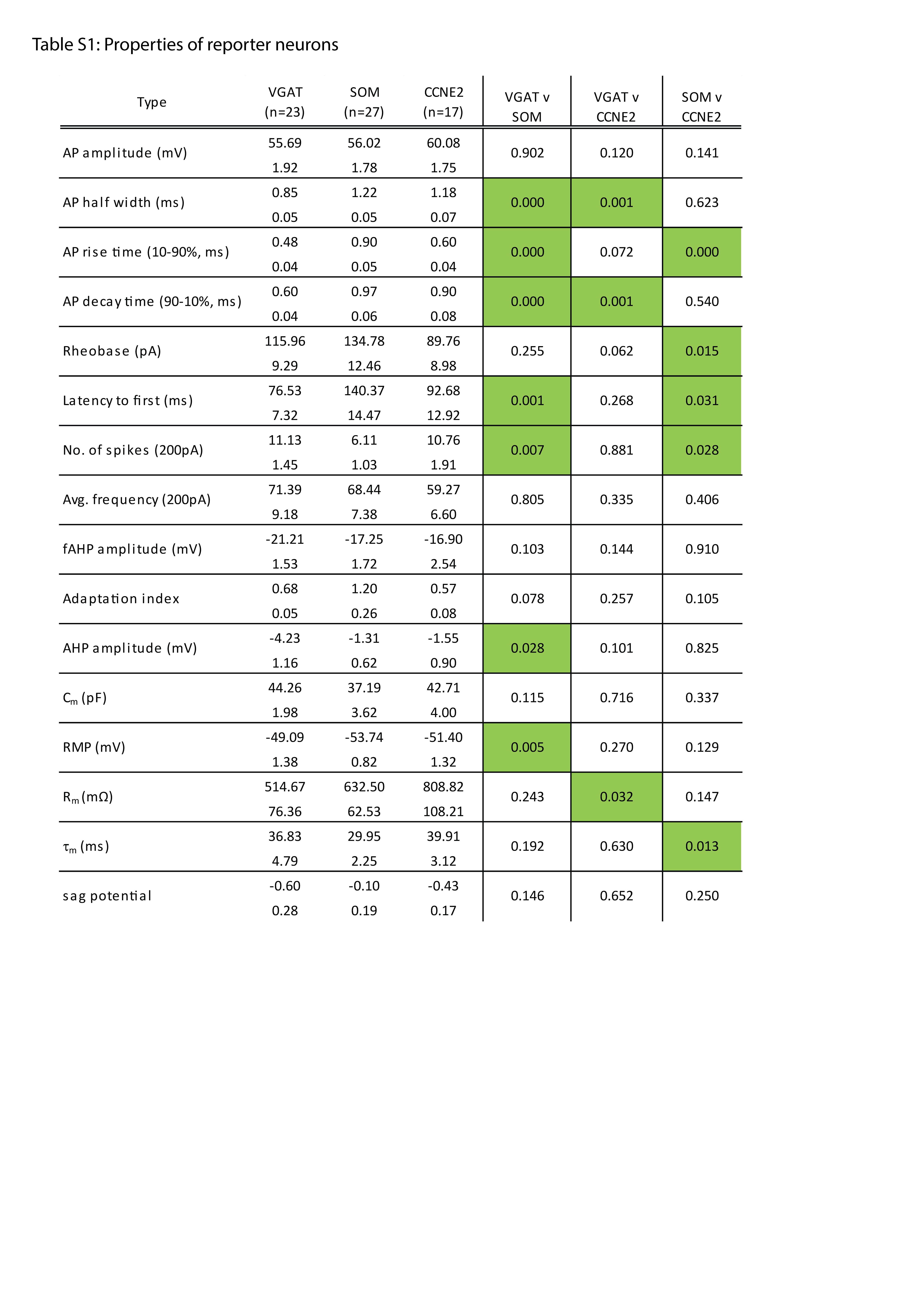

Supplement: Supplementary file 3 — Table S1. Electrophysiological properties of TNc reporter neurons. In each row, averages are represented (top) followed by S.E.M. AP, action potential; (f)AHP, (fast) after‐hyperpolarizing potential; RMP, resting membrane potential; τm, membrane time constant; Rm, input resistance; Cm, capacitance. [file PHY2-7-e14112-s003.png]
